# A Rare Case of Destruction of the First Metatarsophalangeal Joint in a Patient With Gout

**DOI:** 10.7759/cureus.32285

**Published:** 2022-12-07

**Authors:** Vellanki Sravan, Swaroop Solunke, Rushikesh Abhyankar, Vinod Nair

**Affiliations:** 1 Orthopaedics and Trauma, Dr D Y Patil Medical College, Dr D Y Patil Vidyapeeth, Pune, IND; 2 Orthopaedics, Dr D Y Patil Medical College, Dr D Y Patil Vidyapeeth, Pune, IND; 3 Orthopedics and Traumatology, Dr D Y Patil Medical College and Hospital, Pune, IND; 4 Orthopaedics, Dr D Y Patil Medical College and Hospital, Pune, IND

**Keywords:** first metatarsophalangeal joint, gouty tophi, monosodium urate deposition, podagra, gout

## Abstract

Gout is a crystal deposition disorder caused due to the deposition of monosodium urate crystals in joints and other tissues secondary to hyperuricemia. Podagra is the term for gout of the first metatarsophalangeal joint.

In our case report, a 30-year-old male patient came to our OPD with complaints of swelling over the first metatarsophalangeal joint for one year, which was insidious in onset, localized, and had a sudden increase in size over the past three months. The patient also complained of an inability to properly wear his shoe. A plain radiograph was done, which was suggestive of an expansile lesion with the destruction of the first metatarsophalangeal joint and the erosion of the joint surface extending to the head of the first metatarsal and the proximal phalanx of the great toe. Lab investigations revealed a serum uric acid level of 10.2 mg/dl and an acid phosphatase level of 8.92 U/L.

Excision of the lesion was done and a frozen section biopsy was sent intra-operatively which confirmed the presence of monosodium urate crystals. A fibular strut graft was taken to fill the defect using a square nail passing through the first metatarsophalangeal joint and a Kirschner wire was added to the interphalangeal joint to maintain the stability of the reduction. The foot was immobilized for six weeks following which the Kirschner wire was removed and range-of-motion exercises started. There was no residual deformity, and the patient responded well to the treatment.

## Introduction

The term "gout" is used to describe a diverse group of conditions affecting only humans, usually secondary to hyperuricemia [1]. Uric acid is formed as the end product of purine metabolism in the liver. Purine-rich foods contribute to urate load in the body, however, the main cause of hyperuricemia is impaired excretion. A serum urate value of more than 7 mg/dl is considered saturated, and symptoms occur above this level in most of the patients [2]. Two-thirds of uric acid is excreted by the kidneys and the remaining by the intestines. In the kidneys, it is filtered and secreted and 90% is reabsorbed. Humans lack the enzyme uricase present in other mammals, which converts uric acid to allantoin, which is a more water-soluble form [3]. As the uric acid level increases, it gets accumulated in blood and soft tissues. When tissues are saturated with uric acid, it precipitates as crystals in joints. Precipitation is enhanced by acidic and cold environments leading to increased deposition of crystals in peripheral joints, the most common being the first metatarsophalangeal joint [3]. Podagra is the term used for uric acid crystals precipitating in the first metatarsophalangeal joint in a patient with gout.

Clinically, most patients who have an increased uric acid level are asymptomatic and no treatment is needed. The patient may have a history of alcohol consumption, specifically beer, and a purine-rich diet. Patients with co-morbidities like hypertension and hyperlipidemia are more susceptible to developing symptoms. The patient usually seeks medical attention with complaints of a red hot swollen joint, most commonly at the base of the great toe [4]. Joint aspiration for evaluating the uric acid crystals is done to confirm the diagnosis. These crystals look negatively birefringent under polarized microscopy.

Treatment is necessary for only symptomatic patients with daily activities affected due to the disease. Urate-lowering medications like Allopurinol, Probenecid, and the most recent Topiroxostat can be used to lower serum uric acid levels [5-7].

## Case presentation

**Figure 1:**
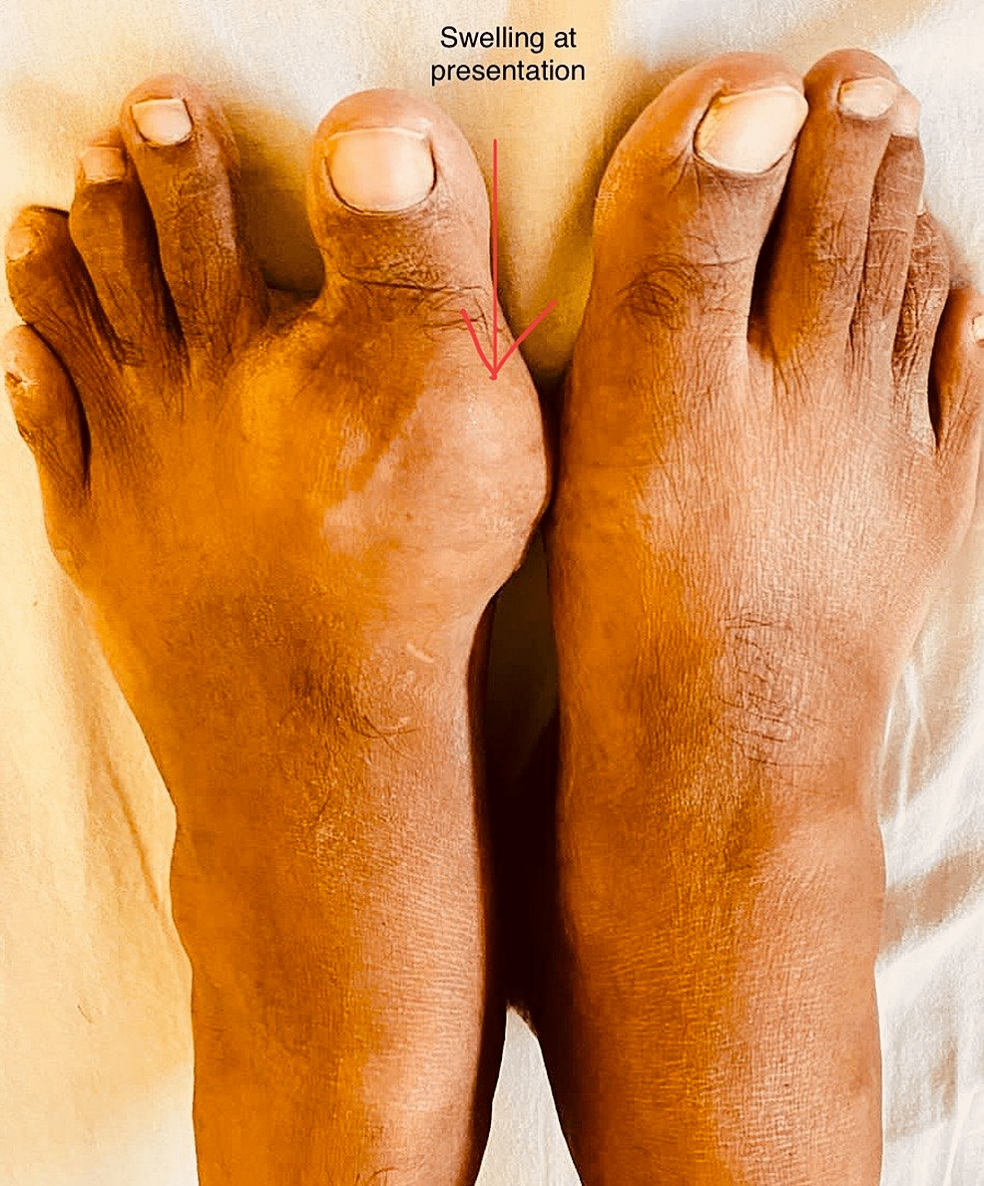
Clinical picture of the foot at the time of presentation

A 30-year-old male patient came to our OPD with swelling over the first metatarsophalangeal joint for one year, which was insidious in onset, localized, and had increased in size over the past three months as shown in Figure 1.

**Figure 2:**
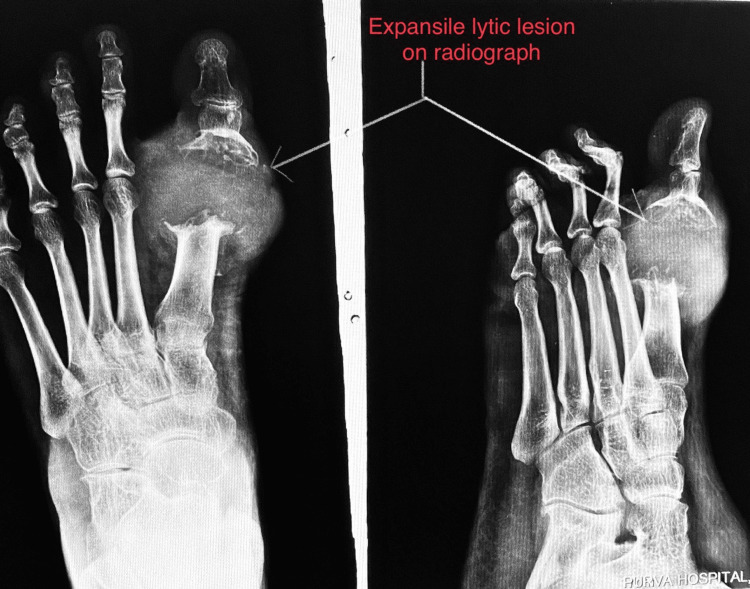
Preoperative radiograph

The patient had difficulty wearing his footwear due to the swelling. The patient had no history of similar complaints in the past. A plain radiograph of the foot was done, which revealed an expansile, lytic lesion eroding the first metatarsophalangeal joint as shown in Figure 2.

**Figure 3:**
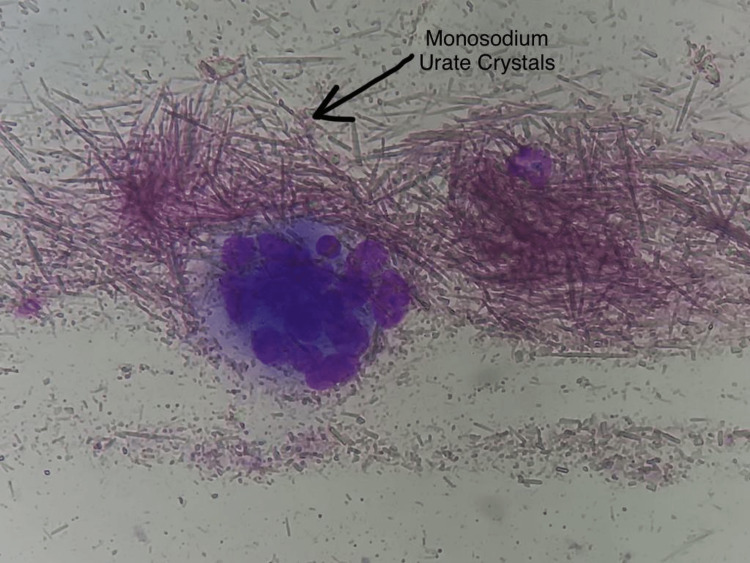
Histopathological presence of monosodium urate crystals on frozen section biopsy

Blood investigations showed an increased serum urate level of 10.2 mg/dL and an increased acid phosphatase level of 8.92 U/L. Excision of the lesion was done with the patient under spinal anesthesia using a dorsomedial approach, and a frozen section biopsy was taken and immediately sent for histopathological examination. The histopathological examination revealed the presence of monosodium urate crystals thereby confirming the diagnosis of gout as shown in Figure 3.

**Figure 4:**
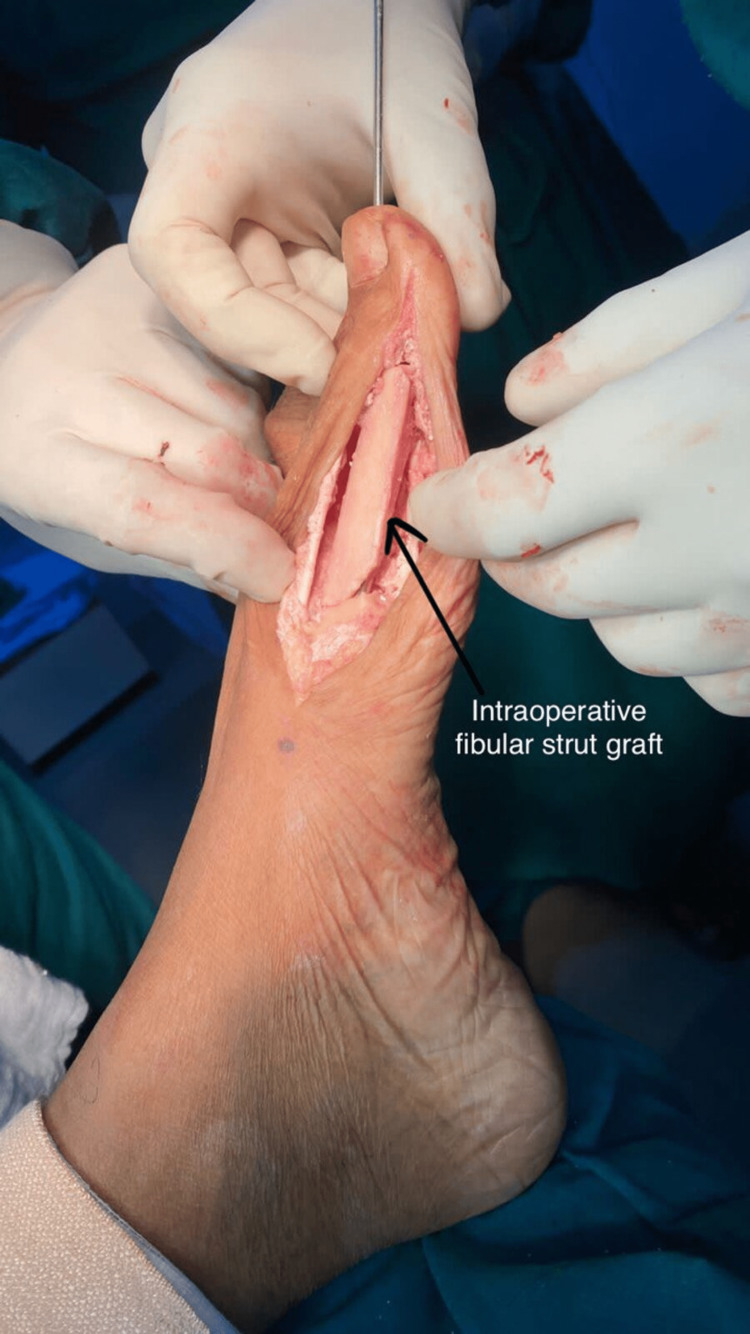
Intraoperative picture after filling the bony defect with fibular strut graft and transfixing with a square nail

The defect of the first metatarsophalangeal joint post-excision was measured, and a fibular strut graft of similar length was harvested using the lateral approach for the fibula. The gap was filled using the graft, which was held in place using a square nail passed through the proximal phalanx into the metatarsal. An additional Kirschner wire was passed to stabilize the inter-phalangeal joint as shown in Figure 4.

**Figure 5:**
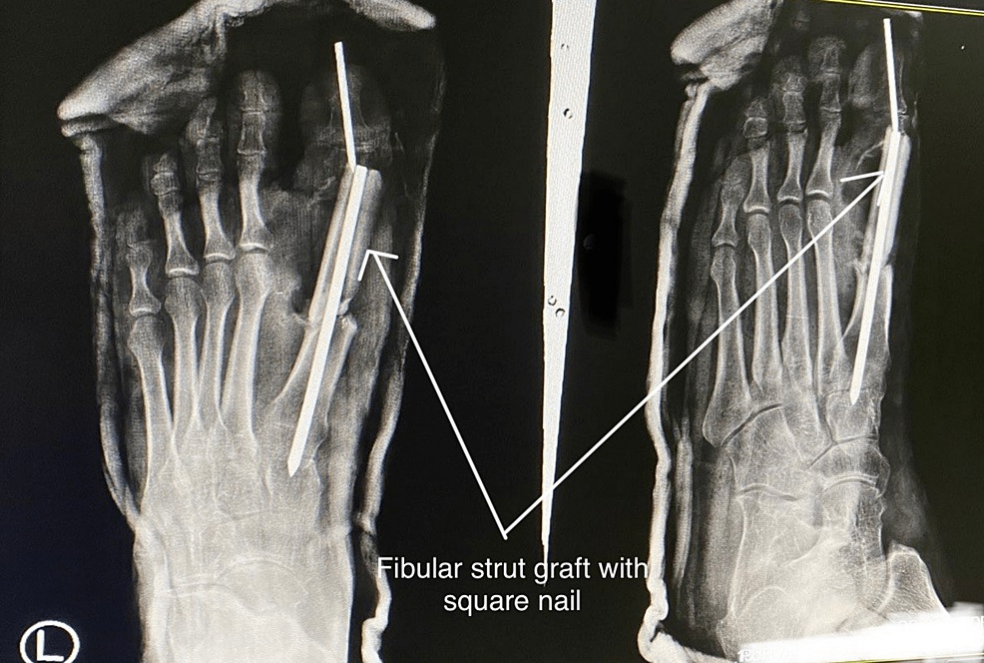
Immediate postoperative X-ray showing a fibular strut graft filling the bony defect fixed with a square nail and Kirschner wire

Routine closure was done and a below-knee slab was given postoperatively to immobilize the joint. A postoperative radiograph was taken as shown in Figure 5.

**Figure 6:**
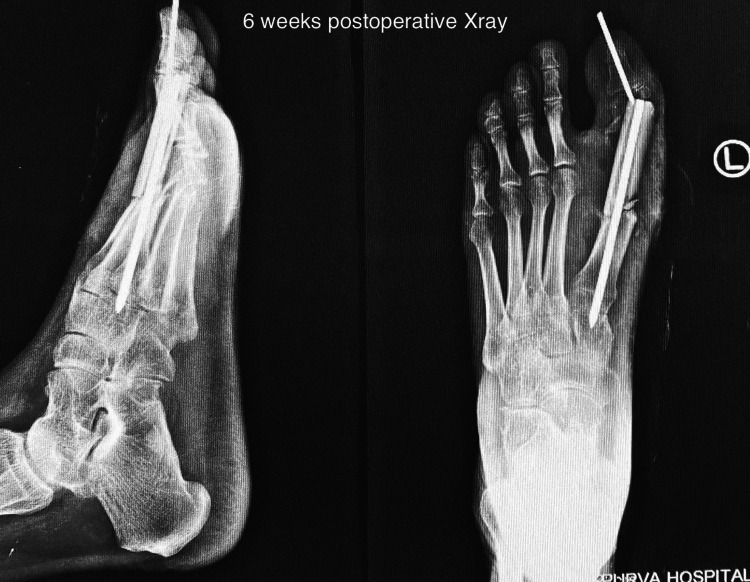
Six weeks postoperative X-ray showing early signs of union

Another radiograph was taken at six weeks' time, which showed signs of union as shown in Figure 6. The Kirschner wire was removed post six weeks and gradual weight bearing was started as tolerated by the patient.

## Discussion

Podagra is the term used for gout of the first metatarsophalangeal joint. Tophus deposition in the joint is a common finding in gout, however, destruction of articular cartilage and surrounding bony erosion is rarely seen. The radiographic picture may suggest a neoplastic growth, especially a giant cell tumor being the most likely differential diagnosis. Thus, histopathological examination is of utmost importance in the diagnosis of these cases.

The majority of the cases of podagra are treated conservatively with uricosuric drugs and often respond well to it. Surgical management is rarely indicated in the presence of bony erosions and cartilage damage.

In elderly patients with podagra, arthrodesis of the first metatarsophalangeal joint is a well-known treatment option [8]. Arthroscopic removal of tophi is also another surgery done to relieve pain in gout [9]. However, in young patients, surgeons face a dilemma between the preservation of the joint and arthrodesis.

In our case, the patient was a young male with an expansile lesion eroding the head of the first metatarsal and the base of the first proximal phalanx of the great toe; preservation of the joint was not an option. Hence, the removal of tophi and reconstruction of the defect with a fibular strut graft was done with the aim to provide a painless joint that does not hinder the daily activities of the patient.

The Masquelet technique is a well-known method of reconstruction of the first metatarsophalangeal joint using a fibular strut graft in cases of osteomyelitis [10]. A similar technique was used in our case to reconstruct the joint after the removal of tophi and lytic bone.

## Conclusions

Podagra is a well-known clinical feature of gout with tophi deposition in the first metatarsophalangeal joint. However, a lytic lesion along with the erosion of the adjacent bone is a rarely-seen complication of the disease. Hence, the removal of tophi and reconstruction of the joint is necessary to provide a pain-free and weight-bearing first metatarsophalangeal joint.
